# Effect of *Helichrysum italicum* in Promoting Collagen Deposition and Skin Regeneration in a New Dynamic Model of Skin Wound Healing

**DOI:** 10.3390/ijms25094736

**Published:** 2024-04-26

**Authors:** Diletta Serra, Sara Cruciani, Giuseppe Garroni, Giorgia Sarais, Fikriye Fulya Kavak, Rosanna Satta, Maria Antonietta Montesu, Matteo Floris, Carlo Ventura, Margherita Maioli

**Affiliations:** 1 Department of Biomedical Sciences, University of Sassari, Viale San Pietro 43/B, 07100 Sassari, Italy; dilettaserra9@gmail.com (D.S.); sara.cruciani@outlook.com (S.C.); giugarroni21@gmail.com (G.G.); fikriyefulyakavak@gmail.com (F.F.K.); matteo.floris@gmail.com (M.F.); 2 R&D Laboratory Center, InoCure s.r.o, Politickych veziu 935/13, Nové Mesto, Praha 1, 110 00 Prague, Czech Republic; 3 Department of Life and Environmental Sciences, University of Cagliari, University Campus, 09042 Cagliari, Italy; gsarais@unica.it; 4 Department of Medical, Surgical and Experimental Sciences, University of Sassari, 07100 Sassari, Italy; rsatta@uniss.it (R.S.); mmontesu@uniss.it (M.A.M.); 5 Laboratory of Molecular Biology and Stem Cell Engineering—Eldor Lab, Istituto Nazionale di Biostrutture e Biosistemi (INBB), 40128 Bologna, Italy; carlo.ventura@unibo.it; 6 Center for Developmental Biology and Reprogramming—CEDEBIOR, Department of Biomedical Sciences, University of Sassari, Viale San Pietro 43/B, 07100 Sassari, Italy

**Keywords:** stem cells, fibroblasts, wound healing, *Helichrysum italicum*, tissue regeneration, bioreactor, molecular mechanisms, dynamic cultures

## Abstract

Natural products have many healing effects on the skin with minimal or no adverse effects. In this study, we analyzed the regenerative properties of a waste product (hydrolate) derived from *Helichrysum italicum* (*HH*) on scratch-tested skin cell populations seeded on a fluidic culture system. *Helichrysum italicum* has always been recognized in the traditional medicine of Mediterranean countries for its wide pharmacological activities. We recreated skin physiology with a bioreactor that mimics skin stem cell (SSCs) and fibroblast (HFF1) communication as in vivo skin layers. Dynamic culture models represent an essential instrument for recreating and preserving the complex multicellular organization and interactions of the cellular microenvironment. Both cell types were exposed to two different concentrations of *HH* after the scratch assay and were compared to untreated control cells. Collagen is the constituent of many wound care products that act directly on the damaged wound environment. We analyzed the role played by *HH* in stimulating collagen production during tissue repair, both in static and dynamic culture conditions, by a confocal microscopic analysis. In addition, we performed a gene expression analysis that revealed the activation of a molecular program of stemness in treated skin stem cells. Altogether, our results indicate a future translational application of this natural extract to support skin regeneration and define a new protocol to recreate a dynamic process of healing.

## 1. Introduction

The skin is the most important protective barrier between the inside of the body and the outside world. Sustaining the physiological healing process, as would be desirable in case of a wound, represents a major goal in regenerative medicine [1]. Skin cells produce a healthy environment composed of an extracellular matrix (ECM), specific peptides, and growth factors [2]. The cellular and biochemical events that take place during the wound repair process are divided into four phases: hemostasis, inflammation, proliferation, and tissue remodeling [3]. This process involves the spatial and temporal cooperation of different cell types with distinct roles and secreted molecules [4], able to preserve the barrier function of the skin [5]. The remodeling phase is characterized by the deposition of collagen, the replacement of the damaged site with fresh tissue, and, finally, the formation of scar tissue [6]. The tissue slowly increases in strength and flexibility and the collagen is remodeled from type III to type I until complete wound closure is achieved [6]. 

The inner part of the epidermis, consisting of the basal cell layer, comprises different stem cell (SC) populations. Stem cells are usually named according to the niches of the skin in which they are located. However, they are collectively known as skin stem cells (SSCs) [7]. These cells provide the skin’s regenerative capacity through extensive proliferation and differentiation. This requires careful symmetric and asymmetric cell division to maintain the stem cell pool [8]. Increased attention has been directed to SSCs for their role in wound healing, although emerging therapeutic approaches are considering the use of mesenchymal stem cells (MSCs) [9]. According to recent research, MSCs are considered the best seed cells for tissue engineering [10,11]. They can be isolated from different tissues, such as skin, bone marrow, adipose tissue, the umbilical cord, and dental pulp. During tissue injury, these cells undergo a strong self-proliferation, showing multidirectional differentiation and powerful secretion skills. Furthermore, MSCs have immunomodulatory functions, thus creating a helpful inflammatory microenvironment in the damaged area [12,13]. The employment of MSCs in regenerative medicine allows a supportive immune setting along with the secretion of growth factors able to promote endogenous tissue healing [13]. These cells are able to affect different cell behaviors, together with all stages of wound healing [4,14]. MSCs are undifferentiated cells able to replace damaged components and restore tissue function [15]. Actually, these cells promote skin cell migration, angiogenesis, re-epithelialization, and granulation tissue formation to support a regenerative environment [4,16,17,18]. The behavior of stem cells is influenced by their position. After injury, niche factors and environmental changes produce regenerative signals able to increase stem cell activities and preserve tissue homeostasis [19]. Any disarrangement in stem cell function and the biological activity of growth factors results in delayed wound healing or the creation of hypertrophic scars [20]. Furthermore, skin fibroblasts show a multifunctional role during the wound healing process, as their morphological plasticity can lead to changes in myofibroblasts. This transition supports the fibrotic mechanism and wound contraction capability [21]. Fibroblast differentiation and proliferation preserve the epithelial barrier from damage, increasing the release of collagen and elastin [21,22,23]. Collagen is a fibrillar protein of the extracellular matrices and connective tissues, widely used in the cosmetics industry for its physical properties [24,25]. Proteolytic degradation of native collagen releases polypeptide fragments that present chemotactic properties, inducing cell differentiation, migration, and recruitment, all useful for activating the healing process [25,26].

In recent years, new cell culture systems have been developed to create a continuous dynamic environment capable of mimicking in vivo cell growth conditions and promoting cell proliferation and differentiation [27]. Biochemical stimuli influence morphogenesis through bi-directional coordinated interactions affecting the metabolism and development of many healthy and cancerous human cells. Indeed, mechanotransduction mechanisms guide cell development, homeostasis, and regeneration [28]. Within this context, several bioreactors have been developed to provide native physical stimuli in vitro using hydrostatic pressure [29].

Indeed, several studies have shown that fluid flow induces forces able to influence stem cell differentiation [30,31]. Cells cultured under this condition are exposed to a constant stimulus enhanced by nonstop perfusion of the culture medium. This evidence shows that a bioreactor design that generates a microenvironment with appropriate biochemical and biomechanical signals could promote the development of a dynamic culture system [32].

In adulthood, tissue healing becomes progressively more critical, as stem cell activity is influenced by cellular senescence and stem cell niches may be damaged [33]. Octamer-binding transcription factor 4 (Oct-4) is essential for the maintenance and induction of stem cell pluripotency, along with other factors such as sex-determining region Y-box 2 (Sox2) and homeobox protein NANOG [34]. The expression of these stemness genes regulates the role of SSCs in self-renewal and differentiation [35]. Senescence is a process in which cells lose their ability to proliferate, resulting in cellular aging. In this context, the senescence of stem cells must be prevented [36]. Cellular senescence is involved in the pathogenesis of chronic wounds. Consequently, incomplete healing and reduced tissue integrity are caused by the presence of senescent cells in the wound environment, characterized by a reduced self-preservation and proliferation [37]. Within this context, Bmi1, a Polycomb group repressor, has been shown to be necessary for stem cell self-renewal. Nevertheless, Bmi1 maintains the stem cell pool by preventing premature senescence, directly by repressing genes involved in senescence or potentially by inducing telomerase activity to prevent telomere shortening [38].

Although several therapeutic procedures based on the use of antibiotics, antiseptics, and anti-inflammatory agents are available for wound management, the use of medicinal plants and natural products as the main source of wound healing compounds has emerged in recent decades [39]. The use of herbal medicines exhibits greater safety with more accessible costs as compared to chemical drugs [40].

Natural compounds display beneficial therapeutic effects, both on skin diseases and on restoring skin homeostasis [41]. The identification of bioactive molecules in plants offers an opportunity to use these products to enhance tissue regeneration and wound treatments [42]. Within this context, *Helichrysum italicum* (*HH*) is a useful source of natural bioactive compounds, among which terpenes and phenolics are the most representative. The traditional use of this plant includes the treatment of allergies, colds, coughs, skin, liver and gallbladder disorders, inflammation, infections, and insomnia [43]. As previously reported, *HH* is rich in phytochemicals, especially caffeoylquinic acid derivatives and naringenin derivatives [44]. *HH* consists of these different sets of compounds that can be obtained by different separation methods, including steam distillation. Distillation products of essential oils, called “hydrolates”, are studied in natural cosmetics/biomedicine for their healing effects on the skin [45]. For these reasons, hydrolates can be used as essential oils for skin treatment [46]. In addition, they have not shown any clinical sign of skin irritation, making them safe for consumers as potential wound healing agents and explaining their popular therapeutic indication [47].

Using suitable tools in vitro able to reproduce the tissue environment in vivo will unravel the behavior and the different cellular mechanisms involved in the skin regenerative process. The qualitative and quantitative features of *HH* in the regenerative context have already been analyzed using a static in vitro culture system [44]. Here, we reproduced the same treatment conditions on SSCs and HFF1s in a dynamic system using fluidic cellular crosstalk. For this purpose, in the present study, we used a bioreactor with culture medium perfusion to recreate the physiological interactions occurring in vivo between skin cells. We developed a new protocol of a scratch assay to create a dynamic model of wound healing in vitro. The aim of this work was mainly to compare the regenerative properties of *HH* in a dynamic system versus the results already obtained in static culture. For this reason, we analyzed the role of *HH* in increasing collagen deposition, both in static and dynamic culture conditions. 

## 2. Results

### 2.1. Dynamic Culture Condition Increases Collagen Deposition

**Figure 1:**
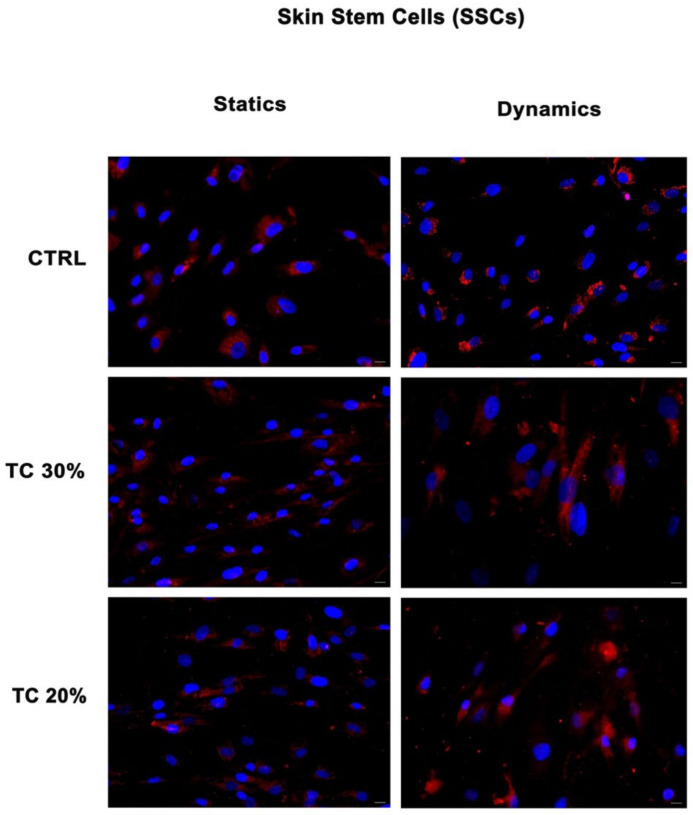
Analysis of collagen deposition in SSCs during wound healing. Immunohistochemical analysis of the expression of collagen type I (red) was assessed in SSCs after scratch assay and treatment with two different concentrations of *HH* (30% and 20%). Control cells (CTRLs) were maintained in basic growing medium after scratch assay. Nuclei are labelled with 4,6-diamidino-2-phenylindole (DAPI, blue). Scale bars: 40 µm, magnification 40×. The figures are representative of different independent experiments.

**Figure 2:**
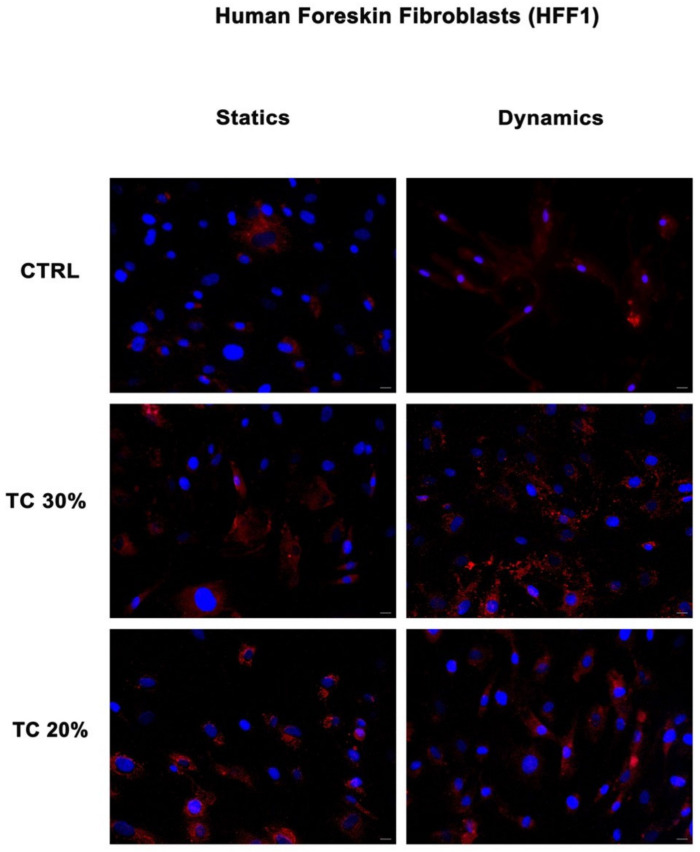
Analysis of collagen deposition in HFF1s during wound healing. Immunohistochemical analysis of the expression of collagen type I (red) was assessed in HFF1s after scratch assay and treatment with two different concentrations of *HH* (30% and 20%). Control cells (CTRLs) were maintained in basic growing medium after scratch. Nuclei are labelled with 4,6-diamidino-2-phenylindole (DAPI, blue). Scale bars: 40 µm, magnification 40×. The figures are representative of different independent experiments.

Figure 1 and Figure 2 show the expression of collagen I in SSCs and human foreskin fibroblasts (HFF1s) exposed to two different concentrations of *HH* (20% and 30%) after a scratch assay as compared to untreated control cells. Collagen deposition was higher in cells treated with the extracts (TC 30% and TC 20%) as compared to untreated control cells (CTRLs) in both HFF1s and SSCs. Furthermore, when comparing static or dynamic cultures, it is evident that the continuous flow generated by the bioreactor enhanced the cellular response by increasing collagen secretion, as compared not only to the untreated controls, but also to the same treatment under static conditions. Also, for the controls, culturing cells under dynamic conditions increased their reparative potential, as compared to the untreated control cells grown in static cultures. For these reasons, subsequent experiments were performed under dynamic conditions.

### 2.2. HH Treatment Regulates Gene Expression in SSCs

**Figure 3:**
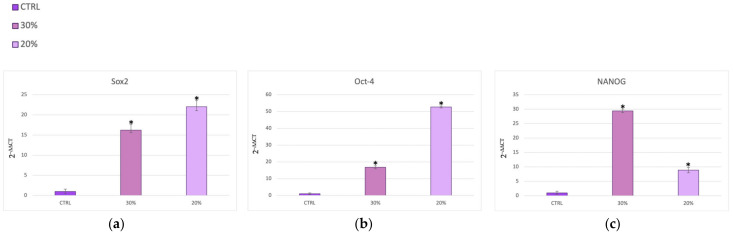
Gene expression analysis of Sox2 (**a**), Oct-4 (**b**), and NANOG (**c**) in SSCs cultured in the presence of the different concentrations of *HH* after the scratch assay. The expression of each gene was normalized to GAPDH and plotted as the fold change (2^−∆∆Ct^) relative to the mRNA expression of the untreated control (CTRL); * *p* value ≤ 0.05.

**Figure 4:**
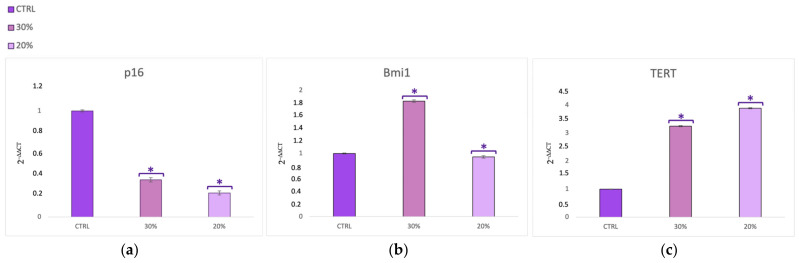
Gene expression analysis of p16 (**a**), Bmi1 (**b**), and TERT (**c**) in SSCs cultured in the presence of the different concentrations of *HH* after the scratch assay. The expression of each gene was normalized to GAPDH and plotted as the fold change (2^−∆∆Ct^) relative to the mRNA expression of the untreated control (CTRL); * *p* value ≤ 0.05.

Figure 3 and Figure 4 show SSCs cultured in the presence of two different concentrations of *HH* (20% and 30%) after the scratch assay. The cells exhibited significant increased expression of Oct-4 (Figure 3a), SOX2 (Figure 3a), Oct-4 (Figure 3b), and NANOG (Figure 3c) when cultured in the presence of 20% and 30% *HH*, as compared to untreated control cells (CTRLs). Figure 4a shows an inhibition of the senescence-regulating gene p16 for both concentrations of *HH* used. On the other hand, Figure 4 reveals significantly higher expression of the Bmi1 (Figure 4b) and TERT (Figure 4c) genes in the presence of both 20% and 30% *HH*, as compared to the untreated controls (CTRLs), with 30% *HH* eliciting the most pronounced stimulatory response.

## 3. Discussion 

A wound is a physical damage that destroys the epithelial barrier of the skin. The application of natural products in wound healing is a traditional practice used to recreate healthy tissue, without adverse effects [41]. Tissue regeneration involves a sequence of cellular events that promote cell migration and collagen deposition [48]. To this end, fibroblasts play an essential role during the early inflammatory phase and the final repair process, stimulating the production of extracellular matrix (ECM) [48]. In vitro, assays evaluating cell migration are useful for analyzing cell migration during wound healing [49]. The scratch assay creates a gap in the confluent monolayer of cells to simulate a wound. The in vitro protocol is based on a few steps: cell culture preparation, a scratch wound assay, and data acquisition and analysis, as previously described [44,50,51]. Plants and their waste products, produced by green practices, can stimulate stem cell proliferation, promoting the restoration of damaged tissue. Most of their bioactive molecules are used in vitro to affect specific culture media and activate the regenerative role of stem cells [52]. 

In the present study, we used a bioreactor to co-culture skin stem cells (SSCs) and fibroblasts (HFF1s), and we compared the effects elicited by a hydrolate of *Helichrysum Italicum* in a scratch model of wound healing in vitro (Supplementary Figures S1 and S2) with the responses obtained under static culture conditions. Dynamic cell and tissue culture has a direct impact on the composition, morphology, and mechanical properties of engineered tissues grown in mechanically stimulated environments [53]. The effects of dynamic media enhance the function of dynamic bioreactors based on flow as compared to static culture systems that are based on diffusion [54]. A bioreactor is a system that reproduces physiological environments, useful for testing cells and tissues as they are structured in vivo [55]. The functions of bioreactors include the supply of suitable nutrients to the cells, the removal of waste, gas replacement, temperature regulation, and the stimulation of mechanical force [56]. There are different types of bioreactors with different sizes, complexities, and functional capabilities. One of the most used bioreactors in tissue engineering applications is characterized by perfusion systems [57]. This type of bioreactor creates a hydrodynamic flux able to induce the cellular response as cell migration, proliferation, and extracellular matrix production [57]. The flow rate generated by the bioreactor recreates a native microenvironment, simulating typical cell communication, as represented in figure in Section 4.4 [18]. We exposed SSCs and HFF1s to different concentrations of *HH* (20% and 30%) after a scratch assay. The immunohistochemical results (Figure 1 and Figure 2) showed not only that *HH* increased collagen deposition, but provided evidence that cells cultured within the bioreactor exhibited an increased reparative potential as compared to cells cultured in static cultures, both in the absence and presence of *HH*. At the same time, our results indicate that *HH* can accelerate epithelization and increase wound contraction by acting on the matrix deposition (Supplementary Figures S4 and S5). Epigenetic modifications repress the expression of p16 in the basal layer of the skin, protecting stem cells from senescence and preserving the tissue regenerative capability [43]. Interestingly, in our experiments, we revealed a downregulation in the expression of this senescence-associated gene in SSCs treated with both *HH* concentrations used as compared to control cells (Figure 4a). On the other hand, Figure 3 shows that *HH* treatment increased the transcription of stemness-associated genes such as Sox2 (panel a), Oct-4 (panel b), NANOG (panel c). The overexpression of these stemness markers preserves the plasticity of stem cells and their ability to regenerate. Conversely, a decrease in their activity during the senescence process has been shown to hamper regeneration in damaged tissues [58,59]. Several studies demonstrate that Bmi1 also regulates the proliferation activity of stem cells and plays a crucial role in the cell cycle and senescence [60]. Bmi1 is downregulated when cells meet their senescence fate [60], allowing the overexpression of the p16 gene. Interestingly, our results showed a high expression of Bmi1 (Figure 4b) with a contemporary downregulation of p16 (Figure 4a). At the same time, Bmi1 expression is related to an increased transcription of Sox2, Oct-4, and NANOG (Figure 3a–c). TERT is downregulated during cellular aging, but it is highly expressed in proliferating cells [61]. Intriguingly, TERT showed a higher expression in cells cultured in the presence of both *HH* concentrations when compared to untreated cells (Figure 4c, Supplementary Figure S3). The finding that such an effect occurred concomitantly with an overexpression of Bmi1, which is recognized as an important positive regulator of TERT, suggests that *HH* may act transcriptionally to counteract cell senescence and possibly telomere shortening. The molecular mechanisms involved in the healing process, unraveled by us, elicited by a waste product, represent a sustainable alternative to conventional dressings. Among medicinal plants, *Helichrysum italicum* (*H. italicum*) is known for its polyphenolic content and for its capability to accelerate skin regeneration and to decrease wrinkles [62,63]. The benefits of hydrolates are related to the presence of polar or partially miscible aqueous volatiles. Due to the chemical features of these compounds, they show antioxidant, antimicrobial, and regenerative effects [64]. Hydrolates are well tolerated by the skin and can be used as an additive to enhance antioxidant activity [64,65]. The chemical composition of the hydrolate obtained by steam distillation of the flowering tops of *Helichrysum italicum* subsp. *Michrophyllum* (*HH*) has already been described [44]. The characterization of the *HH* phenolic compounds shows that caffeoylquinic acid derivatives (chlorogenic acid) were the most abundant compounds, followed by naringenin derivatives [44]. Chlorogenic acid (CA) bears many biological activities, such as antioxidant effects, liver and kidney protection, antibacterial effects, anti-cancer effects, the regulation of glucose and lipid metabolism, anti-inflammatory effects, nervous system protection, and action on blood vessels [66]. Recent research has shown that CA has promising potential in treating wound healing and promoting the synthesis of collagen and elastin [67]. Similarly, naringenin carries different pharmacological properties, such anti-inflammatory, antioxidant, antifibrotic, neuroprotective, antibacterial, and antitumor activities [66]. In skin diseases, different technologies or methods are combined to improve the clinical applications of naringenin due to its low affinity for water [68]. The use of these compounds in the biomedical field requires further investigation to learn more about their safety, efficiency, administration, and bioavailability in humans [68]. Nevertheless, plants, rich in different compounds, are becoming a good source for novel products with different properties [69]. 

The analysis performed on the *HH* tested shows that these waste products can promote regenerative processes without showing a cytotoxic effect on cell proliferation, especially at low concentrations (Supplementary Figure S6). Furthermore, the phytochemical assay revealed that *HH* is rich in phenolic compounds already known for their role in accelerating cell migration and improving wound closure, such as naringenin and chlorogenic acid [44]. These metabolites carry beneficial properties on skin, including the mitigation of skin disorders and reduced healing time, as already demonstrated [63].

On the whole, our results indicate that *HH* is a suitable candidate in the development of strategies aimed at accelerating epithelization and wound healing, acting at the level of wound contraction and collagen deposition.

## 4. Materials and Methods

### 4.1. Preparation of HH

The hydrolate of *H. italicum* (*HH*) was obtained by steam distillation of the flowering tops of *Helichrysum italicum* subsp. microphyllum (Willd.) harvested at the beginning of June 2021 from a crop grown on the “LaNora Officinali” farm located in the Municipality of Solarussa (Province of Oristano, Sardinia) and characterized as previously described [44].

### 4.2. Cell Isolation and Culturing

Human skin stem cells (SSCs) were obtained from biopsies of adult male and female patients after Ethical Committee approval (Ethical Clearance Nos. 0021565/2018 and 22/03/2018—Commissione Etica CNR). They were isolated and cultured as previously described [50]. The positive selection of stem cells from skin was obtained with magnetic cell sorting using a primary monoclonal anti-c/kit (CD117) antibody (Miltenyi Biotec, Minneapolis, MN, USA). Cells showed positivity for all mesenchymal surface markers (CD73, CD90, and CD105) and were negative for CD31 and CD45, as previously described [50].

Human skin fibroblast 1 (HFF1)(HFF-1(ATCC SCRC-1041)) cells were purchased from ATCC (Manassas, VA, USA) and cultured in a low-glucose Dulbecco’s modified Eagle’s Medium (DMEM) (Life Technologies, Carlsbad, CA, USA) supplemented with 10% fetal bovine serum (FBS Life Technologies), 2 mM l-glutamine (Euroclone, Milano, Italy), and 1% penicillin/streptomycin (Euroclone) [70].

### 4.3. Cell Culturing Conditions

Fibroblasts and skin stem cells were divided in two different groups, as described in Table 1. The first group includes fibroblasts and skin stem cells cultured for 48 h in the presence of *HH* at two different concentrations (30% and 20%) after a scratch assay (TC). The second group includes untreated cells, in the absence of *HH* concentration, that underwent wound healing (CTRL). We obtained the concentration of *HH* as a percentage of volume (*v*/*v*)% and analyzed five different scalar concentrations (40%, 30%, 20%, 10%, and 5%) to exclude potentially cytotoxic concentrations [44].

### 4.4. Set up of Bioreactor

**Figure 5:**
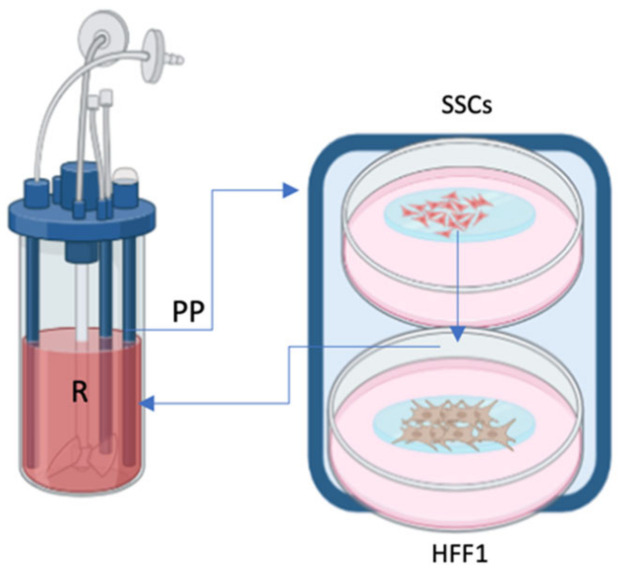
Scheme represents the chamber (Live Box2) and the cells cultured: SSCs (1) and HFF1s (2). The chamber connects to a reservoir of culture medium (R) by a peristaltic pump (PP) (blue lines and arrows in the scheme).

The bioreactor Live flow with the chamber Live Box2 (IVTech, Massarosa, Italy) was set up to recreate in vivo skin layers, as already described [18]. The bioreactor allows culturing different cell types separately while maintaining the crosstalk between them thanks to the culture medium flow. Cells were counted with an automatic cell counter (LUNA, Logos biosystems, Villeneuve d’Ascq, France) and seeded following the isometric proportion of human skin tissue [18]. In the superior layer of the chamber, skin stem cells were seeded in a 0.45 µm membrane (ipCELLCULTURE™, it4ip, Louvain-la-Neuve, Belgium) and subsequently connected with fibroblasts that were seeded on glass in the lower layer. Both were connected to reservoir and peristaltic pump, as represented in Figure 5.

### 4.5. Scratch Assay

After counting, cells were resuspended in 100 μL of complete medium and seeded on glass slides (12 mm Ø) placed in a 24-well multi-well plate until confluence. A scratch test was performed on the cells attached to the glass slides using a pipette tip (200 μL), and afterward they were placed in the different layers of chambers of the Live Box2 according to cell type, as already described [18]. Once the chamber was assembled, it was connected to the LiveFlow pump, and a flow rate of 100 μL/s was set. The bioreactor was placed in an incubator at a temperature of 37 °C and 5% CO_2_ until wound healing was completed. The glass slides with cells were fixed with 4% paraformaldehyde (Sigma Aldrich Chemie GmbH, Hamburg, Germany) for 30 min at room temperature for a post-processing study by confocal microscopy.

### 4.6. Immunostaining

After 48 h of treatment, cells were fixed for 30 min at room temperature (RT) with 4% paraformaldehyde (Sigma Aldrich Chemie GmbH, Germany). After 1 h of permeabilization by 0.1% Triton X-100 (Life Technologies, USA) in PBS at RT, cells were washed in PBS three times for 5 min and incubated with 3% bovine serum albumin (BSA) and 0.1% Triton X-100 in PBS (Life Technologies, USA) for 30 min at RT and then exposed overnight at 4 °C to the primary anti-rabbit anti-Collagen I antibody (Abcam, Cambridge, UK). Finally, cells were washed two times for 5 min in PBS and stained at 37 °C for 1 h in the dark with the fluorescence-conjugated goat anti-rabbit IgG secondary antibody (AF594) (Life Technologies, USA). Nuclei were labelled with 1 µg/mL 4,6-diamidino-2-phenylindole (DAPI). All microscopy analyses were performed with a confocal microscope (TCS SP5, Leica, Nussloch, Germany).

### 4.7. Gene Expression Analysis

Gene expression levels were detected by Real Time-qPCR. SSCs were exposed to the two different concentrations of 30% and 20% *HH* after the scratch test. Total mRNA was isolated using an RNeasy Mini Kit (Qiagen, Hilden, Germany) according to the manufacturer’s protocol. The quantity and purity of RNA were measured by OD 260/280 nm using a Nanodrop (Thermo Fisher Scientific, Waltham, MA, USA). Then, 2.5 ng of RNA from each sample in triplicate was reverse transcribed and amplified by a Luna^®^ Universal One-Step RT-qPCR Kit (New England Biolabs, Ipswich, MA, USA) via the Thermal Cycler (Bio-Rad, Hercules, CA, USA). The RT-qPCR analysis was performed for SSCs for the stemness markers Oct-4, Sox2, and NANOG and for the cell-cycle-related genes p16, Bmi1, and h-TERT. All the primers used were previously described [15,18,34]. The target Ct values were normalized to GAPDH, considered as a reference gene, and mRNA levels were expressed as the fold change (2^−∆∆Ct^) relative to the mRNA levels observed in untreated controls.

### 4.8. Statistical Analyses

The experiments were performed two times with three technical replicates for each treatment. Two-way analysis-of-variance ANOVA tests with Tukey’s correction and the Wilcoxon signed-rank test were used, assuming a *p* value < 0.05 as statistically significant. We considered * *p* < 0.05.

## 5. Conclusions

The present study confirms the regenerative properties of *HH* and promotes the use of natural extracts as a safe skin treatment of wounds without side effects. The healing process was implemented by the bioreactor flow, which re-created the native cellular microenvironment and increased collagen deposition. The specific design of the bioreactor is crucial in studying molecular and physiological cellular changes. Our results confirm that the bioreactor is an essential means in the ability to influence different cellular mechanisms and biological processes. Different types of bioreactors can be used to promote the in vitro development of new tissues by providing biochemical and physical regulatory signals to cells, encouraging them to differentiate and/or produce extracellular matrix [71]. Further in vitro and in vivo studies are needed to translate these results into future applications for tissue regeneration.

## Figures and Tables

**Table 1:** Culture conditions of SSCs and HFF1s undergoing wound healing (− without, + with); T = treatment with *HH*; S = scratched cells.

Experimental Conditions	
CTRL	− Treatment (T)/+ Scratch (S)
Treated Cells (TC)	+ Treatment (T)/+ Scratch (S)

## Data Availability

The data of the current study are available in the manuscript.

## Supplementary Materials

The supporting information can be downloaded at https://www.mdpi.com/article/10.3390/ijms25094736/s1.
