# Supplementary material for: Effect of Helichrysum italicum in Promoting Collagen Deposition and Skin Regeneration in a New Dynamic Model of Skin Wound Healing
Source: Int J Mol Sci. 2024 Apr 26;25(9):4736. doi: 10.3390/ijms25094736 (PMC11083432; doi:10.3390/ijms25094736)
Supplement: Supplementary file 1 [file ijms-25-04736-s001.zip › ijms-2905656-supplementary.pdf]

International Journal of
Molecular Sciences

MDPI

Supplementary Materials

The ability of HH to stimulate fibroblast and stem cell proliferation and/or migration was detected by the scratch assay. Supplementary Figures S1 and S2 and show the migration of cells after scratch for different time point (day 0 and 48h). Fig.1 shows fibroblasts treated with different concentrations of HH (TC 30 % and TC 20 %). Figure S1 revealed that fibroblasts treated with 20 % and 30 % HH show a higher number of migrating cells detectable in the wound site as compared to control cells (CTRL). After 48 hours, samples treated with the selected concentrations of HH (30 % and 20 %) reached confluence. On the other hand, in control cells, the wound site was not healed. Similarly, Figure S2 shows SSCs migration and proliferation induced by 30 % and 20 % HH. After 48h, also the cells treated with 30 % HH showed complete confluence of the wound site. All results were analyzed measuring wound closure area after 48h of HH treatment as compared to control untreated cells.

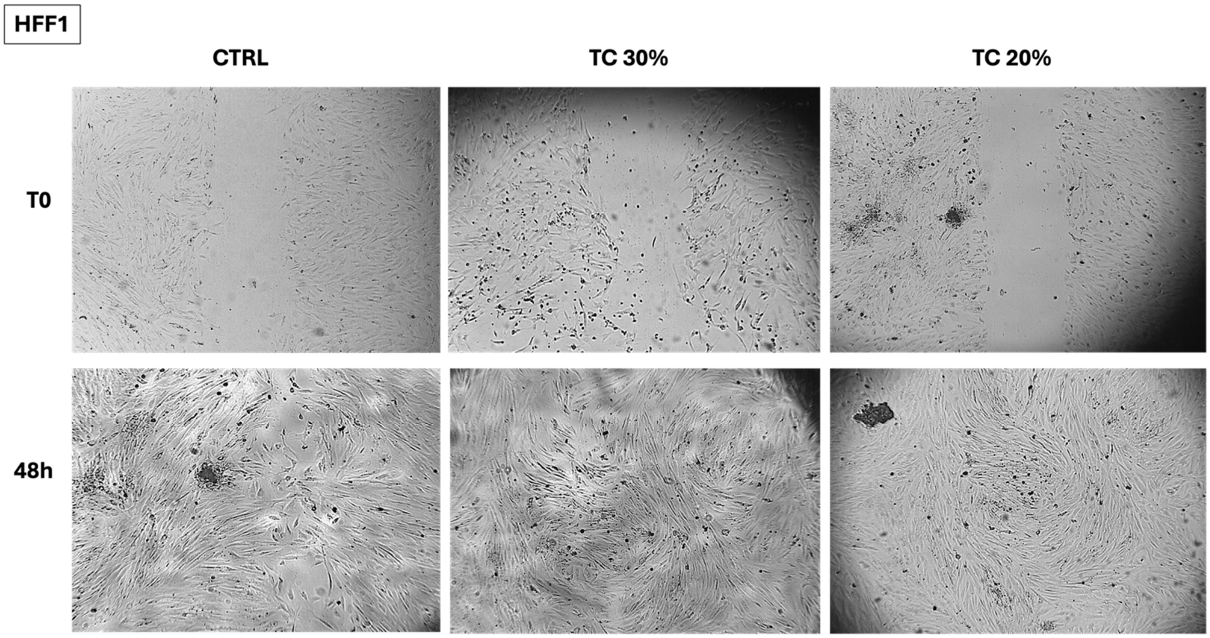

Figure S1. Fibroblast migration and proliferation after scratch and treatment with different scalar dilutions of HH (TC 30% and TC 20%). Images were taken with inverted light microscope at the time of cutting (day 0) and after 48 of treatment.

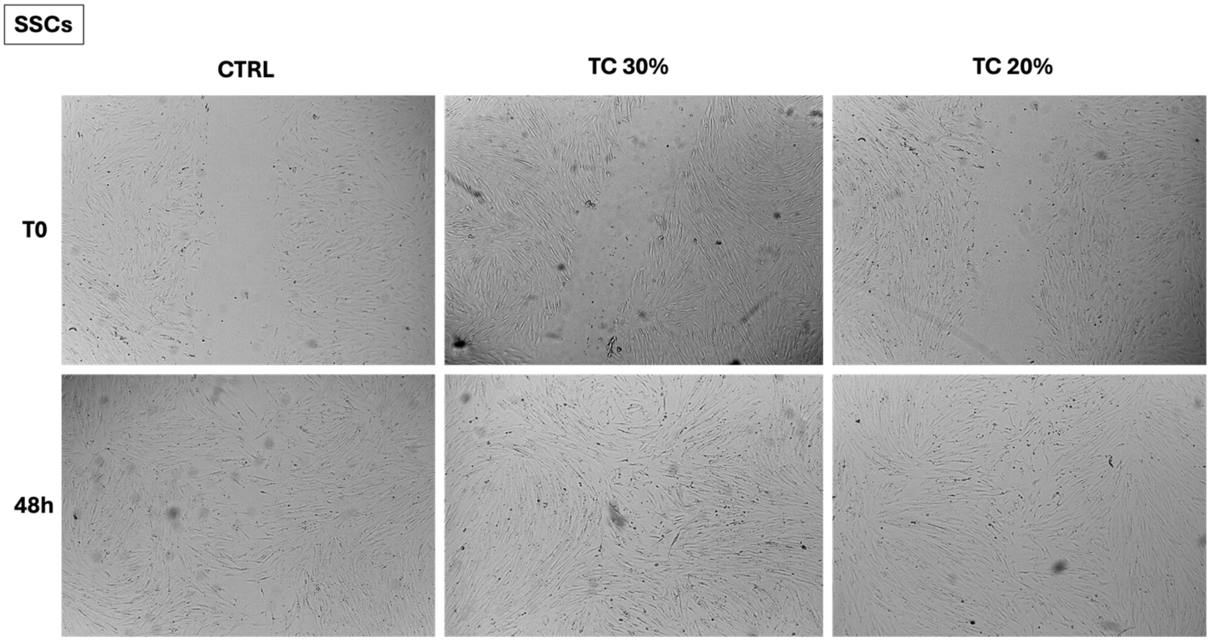

Figure S2. Skin stem cell migration and proliferation after scratch and treatment with different scalar dilutions of HH (TC 30% and TC 20%). Images were taken with inverted light microscope at the time of cutting (day 0) and after 48 of treatment.

Increased levels of TERT expression in presence of both 20% and 30% of HH, as compared to untreated controls (CTRL), where further confirmed by telomerase activity (Figure S3). The presence of both 20% and 30% of HH, on SSCs, increased the enzymatic activity, contributing to counteract cell senescence and telomere shortening.

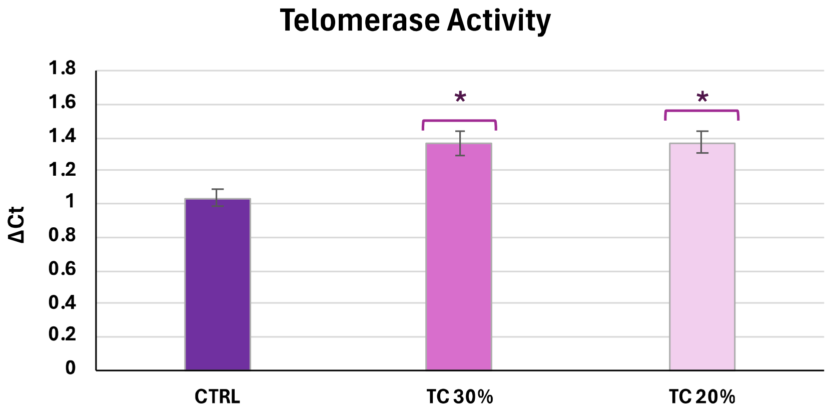

Figure S3. Effect of HH on telomerase activity in SSCs cultured in the presence of the different concentrations of HH after the scratch, as compared to control cells. * p value ≤ 0.05.

Extracellular matrix deposition was assessed by analysis of collagen type I by confocal microscope and by toluidine blue staining in SSCs and HFF1 cultured in the presence of the different concentrations of HH (30% and 20%) after the scratch. Both treatments increased the extracellular matrix deposition, as compared to untreated controls, with a significant increased effect for 20% HH at the end of 48h of treatment.

International Journal of
Molecular Sciences

MDPI

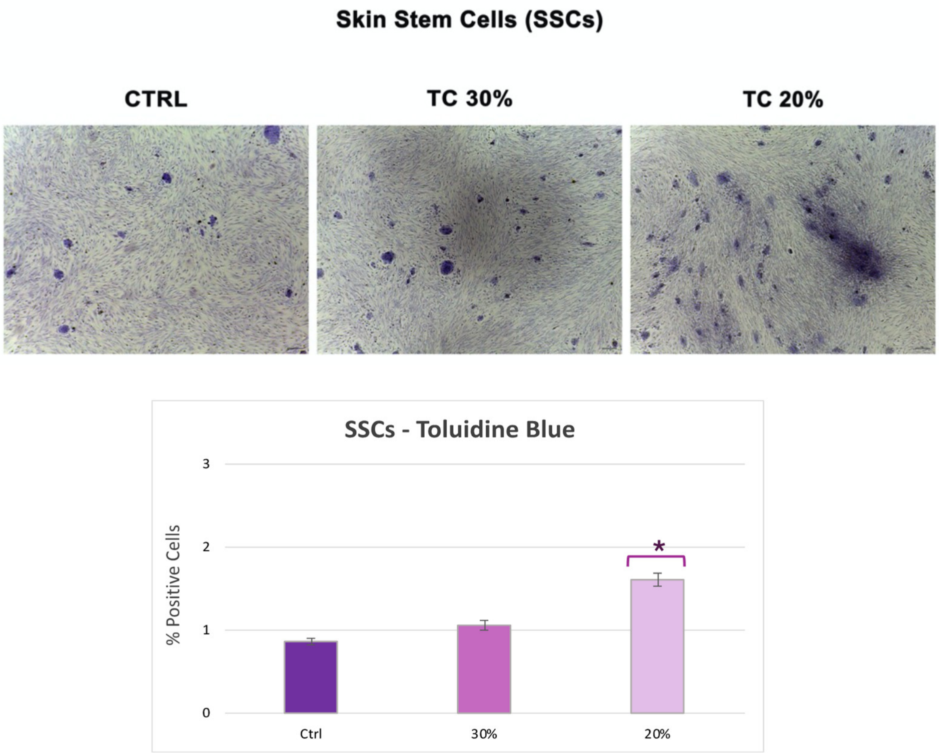

Figure S4. Toluidine blue staining of SSCs cultured in the presence of the different concentrations of HH after the scratch, as compared to control cells. Scale bars: 100 μm, magnification 4x. The figures are representative of different independent experiments.

International Journal of
Molecular Sciences

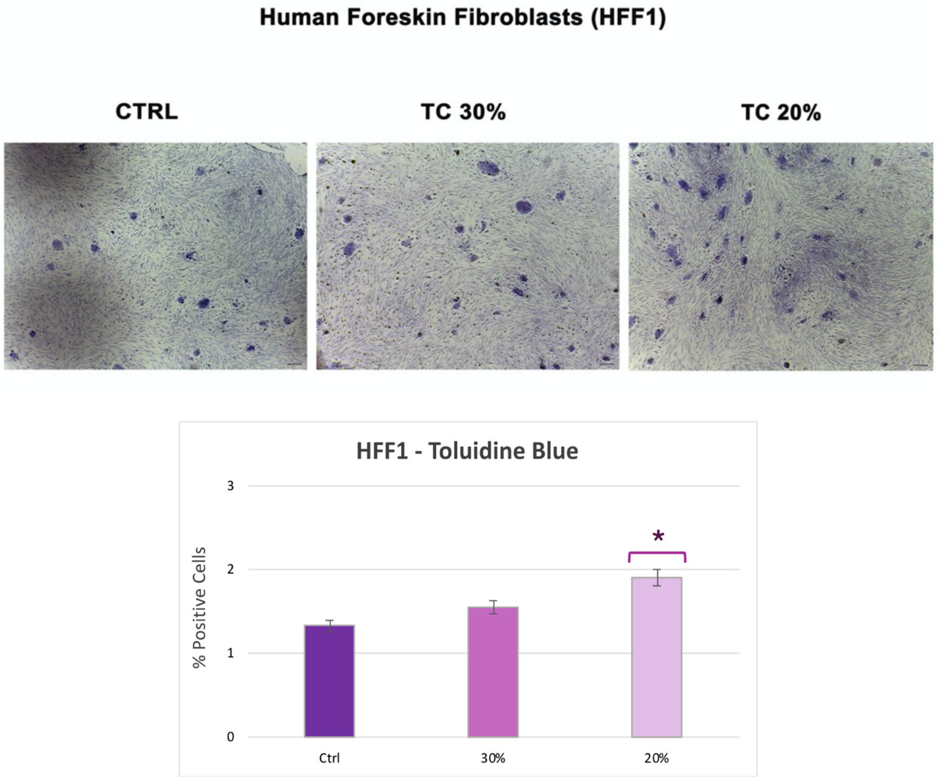

Figure S5. Toluidine blue staining of HFF1 cultured in the presence of the different concentrations of HH after the scratch, as compared to control cells. Scale bars: 100 µm, magnification 4x. The figures are representative of different independent experiments.

Cell proliferation rate was evaluated in HFF1 and SSCs by BrdU assay after treatment with different concentrations of HH (TC 30 % and TC 20 %) at the end of 48h after scratch. During wound healing, a significant increase in cell proliferation for both fibroblasts and stem cells was observed, when cultured with 30 % or 20% HH, as compared to control cells (W-T), that undergo scratch without treatment with HH.

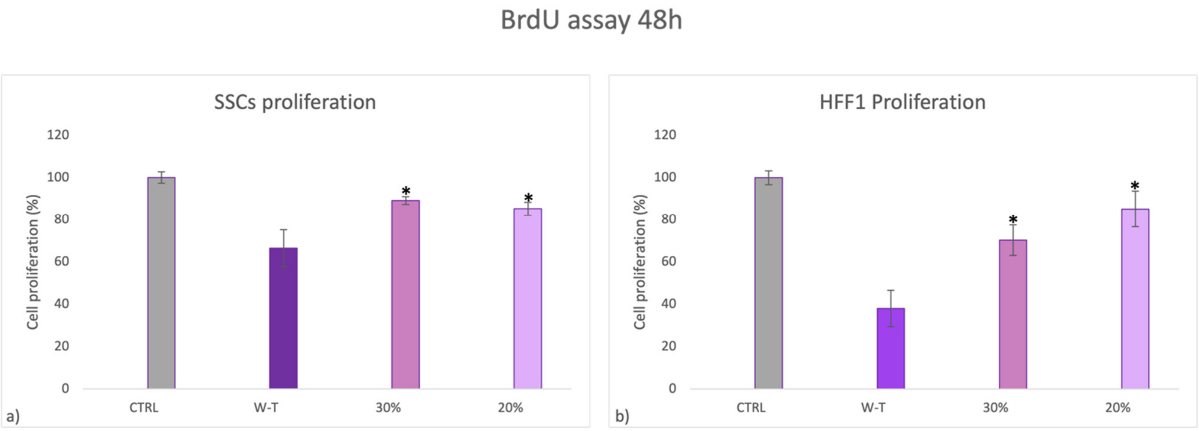

Figure S6. BrdU assay after 48h of treatment with HH in SSCs (panel a) HFF1 (panel b) after scratch test. Cell viability is expressed as percentage to control untreated cells. Data are expressed as mean SD referring to the control * $p < 0.05$.
